# An Indian perspective for umbilical cord blood haematological parameters reference interval

**DOI:** 10.1186/s12887-023-04090-2

**Published:** 2023-06-08

**Authors:** Keyur Sabnis, Swati Ghanghurde, Akash Shukla, Dhriti Sukheja, Mohit V. Rojekar

**Affiliations:** 1 Rajiv Gandhi Medical College, 400605 Thane, India; 2 Department of Pathology, Rajiv Gandhi Medical College, 400605 Thane, India; 3 Department of Biochemistry, Rajiv Gandhi Medical College, 400605 Thane, India

**Keywords:** Haematological parameters, Reference interval, Umbilical cord blood, Peripheral venous blood, Neonates, India

## Abstract

**Background:**

The Haematological Reference Intervals (RIs) are prone to vary on the basis of various factors such as altitude, age, sex, socioeconomic status, etc. These values play a major role in laboratory data interpretation and determine the necessary clinical treatment. Currently, India has no well–established RI for cord blood haematological parameters of newborns. This study aims to establish these intervals from Mumbai, India.

**Method:**

A cross sectional study was conducted in a tertiary care hospital of India from October 2022 to December 2022 on healthy and term neonates having normal birth weight and born to healthy pregnant mothers. About 2 – 3 mL of cord blood was collected from the clamped cord into EDTA tubes from 127 term neonates. The samples were analysed in the haematology laboratory of the institute and the data was analysed. The upper and lower limits were determined using non-parametric method. The Mann–Whitney U test was used to compare the distribution of the parameters between sex of infant, modes of deliveries, maternal age and obstetric history. P value less than 0.05 was considered to declare statistical significance.

**Result:**

The median values and 95% RI for umbilical cord blood haematological parameters of newborns were as follows: WBC = 12.35 [2.56–21.19] × 10^9^/L, RBC = 4.34 [2.45–6.27] × 10^12^/L, HGB = 14.7 [8.08–21.44] g/dL, HCT = 48 [29–67]%, MCV = 109.6 [59.04–159.1] fL, MCH = 34.5 [30.54–37.79] pg, MCHC = 31.3 [29.87–32.75] %, PLT = 249 [16.97–479.46] × 10^9^/L,LYM = 38 [17–62] %, NEU = 50 [26–74] %, EOS = 2.3 [0.1–4.8] %, MON = 7.3 [3.1–11.4], BAS = 0 [0–1]. This study found no statistically significant difference between sex of infants, except MCHC, and obstetric history. A significant difference was observed in WBC, EOS% and absolute NEU, LYM, MON and BAS by delivery type. A higher platelet count and absolute LYM was observed in the cord blood compared to venous blood.

**Conclusions:**

For the first time, haematological reference intervals in cord blood were established for newborns in Mumbai, India. The values are applicable for newborns from this area. Larger study throughout the country is required.

## Introduction

The spread of values which is defined by an upper and a lower reference limit is termed as Reference interval (RI). They are one of the most important elements of a laboratory test result. Physicians compare their patients’ test results and further interpret them on the basis of these intervals. It is hence deemed very necessary that the laboratory community devotes all the sufficient resources in order to ensure that the RI are well–established. It has been observed that most frequently, these intervals represent values for healthy, adult patients and hence, RI for other sets need to be established as in case for newborn, children or pregnant women [1].

The word “normal” has several different connotationions and hence can develop confusion. The terms normal values and normal ranges are obsolete and hence must not be used as a synonym for RI or reference range. To prevent such ambiguities, the terms reference ranges and intervals have been introduced and widely implemented [1].

RI have a major role in the interpretation of laboratory data in patient management i.e. if the laboratory data of the patient is within normal limits or not, clinical trials as well as selection of eligible participants for vaccine trials [2]. These reference values are a subject to change by several factors including age, sex, race, geographical location as well as their dietary pattern. Various studies published globally established the age-related changes occurring in haematological parameters. Significant quantitative as well as qualitative differences in these parameters were exhibited by infants compared to older children and adults [3]. A significant higher value of haematological parameters than those seen in older children and adults were observed in term newborns [4]. It was thus concluded that, it is extremely unsuitable to utilise the reference ranges of adults in order to assess paediatric blood sample [1].

On the other hand, utilising a reference interval obtained from some other geographical location or individuals with a specific age group to a population of interest could potentially lead to inappropriate patient management and futile use of resources [5]. Additionally, individual laboratories determining their own RI often display variations in the final reference intervals that are not supported by analytical or population differences [1].

Some studies confirm that cord blood haemoglobin and newborn weight showed a significant variation due to maternal anaemia [6], even though it has been reported that routine haematological values of newborns have no dependency on the maternal haematological values [7]. Maternal habits like smoking or alcohol consumption, medical problems like diabetes mellitus, eclampsia, hypertension [8] and also the mode of delivery, frequency of pregnancy, obstetrics and abortion history affect the haematological profile of neonates [9]. This kind of variation in the reference intervals on the basis of geographical locations, lifestyle of a person and other above-mentioned factors sets forth the absolute need and rationale to establish reference intervals on consideration of such variations.

Umbilical cord blood collection (UCBC) can be done as a non–invasive method and poses no harm to the mother or the foetus. Other than minor risks involving a delay in the clamping of the cord and its milking that may alter the haematological parameters [10], UCBC is a relatively safe and simple manner of collection of the blood sample. Additionally, studies prove that, a higher sensitivity and accuracy for predicting a disease manifestation of patients by UCBC method against peripheral venous blood collection (PVBC) method concludes that UCBC can be considered as a reliable tool in order to predict a final outcome. Hence, to conduct the current study, we have used the cord blood collection method. This study aimed at providing such intervals for cord blood samples from tertiary care hospital in western part of Maharashtra.

## Material & Methods

### Study design and setting

A cross-sectional study was conducted after due approval from the ICEC in a tertiary care hospital attached to a medical college in western part of Maharashtra, India. The study was conducted for 3 months from October-December 2022. Study participants were recruited from mothers coming to the hospital to get delivery service.

### Sample size determination and sampling technique

Healthy and term neonates (from 37–42 weeks) with normal birth weight born to apparently healthy pregnant mothers who approached our institute to get delivery service were recruited. As per the Clinical Laboratory Standard Institute (CLSI) guideline C28-A3, about 120 samples per partition is required to determine the 95% reference interval [11]. A *priori* selection method was employed, eligible volunteering mothers aged 18 to 45 years were recruited. Mothers with the following conditions were excluded: those with medical conditions like infectious (e.g. Hepatitis B, HIV, Syphilis), chronic illness (e.g. Insulin-dependent diabetes mellitus), obstetric (e.g. preeclampsia), psychological problems and social habits (e.g. smoking, heavily alcohol drinking). Various diagnostic tests (laboratory tests, ultrasound) and history obtained were used to exclude mothers with the listed conditions. On the other hand, mothers who had Hemoglobin (HGB) >  = 12.0 g/dL [12] and inter–pregnancy interval of more than or exactly 18 months as per WHO recommendation [13] were included in the study. Not only this, but also the *posteriori* selection method was used to include eligible neonates of gestational age (37–42 weeks), having 5th minute Apgar score of >  = 7. Babies with respiratory distress, meconium staining, gross congenital anomalies, umbilical cord with true knot, and babies delivered by instrumental delivery were excluded.

### Data collection procedure

All professionals who participated in the collection of the data were oriented about the aim of the study, selection of participants, data confidentiality, safety precautions during collection, transportation, and storage of cord blood samples before the collection of data was initiated. A predesigned questionnaire was utilised in order to assimilate the demographic information and a brief history from all those mothers who had consented for the research and fit into the eligibility criteria. The mothers of neonates gave informed consent after informing them about the aim of study, voluntary participation, confidentiality of the information and their right to withdraw from the study anytime. It is only after the complete separation and expulsion of the placenta from the mother and cutting the umbilical cord post clamping, the cord blood sampling is carried out. The umbilical cord was clamped within 1 min after birth and 2–3 ml of cord blood was collected by operation room (OR) nurse into vacutainers containing EDTA (for complete blood analysis). The sample was well mixed and immediately transported to haematology laboratory for further analysis.

### Screening tests

As a part of the antenatal care (ANC) follow up, all subjects were screened for HIV via antibody tests, Hepatitis B surface antigen and syphilis which are routinely performed for pregnant women. Ultrasonography (USG) was done to rule out any foetal congenital anomalies.

### Quality assurance

The collection of cord blood samples was carried out by experienced OR nurses following the guideline for the cord blood sample collection. A descriptive and informative orientation was given on the proper collection and handling the samples. All procedures that were performed,followedthe standard operating procedures (SOPs).

### Haematological analysis

Complete blood count (CBC) namely white blood cell (WBC), Differential count: neutrophil (NEU#), lymphocyte (LYM#), Basophil (BAS#), Eosinophil (EOS#) and Monocyte (MON#); neutrophil percentages (NEU%), lymphocyte percentages (LYM%), eosinophil percentage (EOS%), Basophils percentage (BAS%), Monocytes percentage (MON%), red blood cells (RBC), hemoglobin (HGB), haematocrit (HCT), mean corpuscular volume (MCV), mean corpuscular hemoglobin (MCH), mean corpuscular hemoglobin concentration (MCHC) and platelets (PLT) were analysed using an automated haematology analyser. In order to investigate the RBC morphology, WBC and PLT abnormalities, peripheral blood smears from cord blood samples were prepared and stained using Wright’s stain.

### Statistical analysis

All the data from the questionnaire and results received from the laboratory were checked for completeness. The collected data was entered in MS Excel and further analysis was done on IBM® SPSS® Statistics. Following the CLSI guide, 2.5^th^ and 97.5^th^ percentiles for haematological and parameters were calculated for 127 neonates of both genders. To compare the distribution of the parameters between sex of infants, delivery modes (other than Instrument assisted delivery), maternal age and obstetric history, the non-parametric Mann–Whitney U test was used. Additionally, statistics were expressed in terms of percentage, ranges, charts and graphs. P value less than 0.05 was considered to declare statistical significance.

Normality of the data distribution was checked with Shapiro and Wilk Test.

## Results

A total of 127 healthy full-term newborns comprising of 68 (53.5%) males and 59 (46.5%) females were enrolled in the study. About 81.3% of the mothers were in the age group 18–30 years, 12% were from outside Mumbai, 100% were married, and 27.56% (35/127) were primiparous. A complete demographic idea has been presented in tabular format in Table 1.

**Table 1:** Sociodemographic and clinical characteristics of study participants (*n* = 127)

Information		Percentage
Resident of	Mumbai	88.19
	Outside Mumbai	11.81
Marital status	Married	100
	Other (unmarried, widowed, etc.)	0
Obstetrics history (I)	Non–primi	72.44
	Primi	27.56
Obstetric history (II)	Abortions	18.9
	No abortions	81.1
Delivery mode	VD	18.12
	CS	81.88
Sex of neonate	Male	53.5
	Female	46.5
Weight of neonate	2.5 – 3.0 kg	78%
	Above 3 kg	22%

There was no statistical significance observed for obstetric history of the mother [primiparous vs multiparous] in any haematological parameter (*p* > 0.05). Significant difference was observed for mode of delivery (*p* < 0.05) (Table 2).

**Table 2:** Independent (2 Groups) Mann–Whitney U Test* of selected haematological parameters by sex of infant, mode of delivery, age of mother and obstetrics history

Parameters	Sex of infant	Mode of delivery	Age of mother	Obstetrics history
WBC	0.575	**0.012**	0.233	0.651
RBC	0.782	0.521	0.703	0.402
HB	0.849	0.691	0.696	0.923
HCT	0.781	0.999	0.87	0.945
MCV	0.802	0.901	0.958	0.91
MCH	0.834	0.404	0.701	0.648
MCHC	**0.016**	0.191	0.29	0.793
PLT	0.739	0.263	0.861	0.445
NEU%	0.213	0.397	0.221	0.953
LYM%	0.482	0.877	0.605	0.228
EOS%	0.2631	**0.013**	0.099	0.625
MON%	0.775	0.396	0.529	0.783
BAS%	0.692	0.37	**0.01**	0.909
NEU#	0.693	**0.004**	0.447	0.876
LYM#	0.302	**0.033**	0.276	0.265
EOS#	0.662	0.784	**0.01**	0.626
MON#	0.3	**0.034**	0.086	0.888
BAS#	0.414	**0.003**	**0.04**	0.316

^*^ *P* values less than 0.05 determines the significance level

A complete summary of sex specific upper and lower intervals for the reference ranges of haematological parameters from umbilical cord blood has be provided in Table 3. Except MCHC, all other parameters are not statistically significant.

**Table 3:** Reference Intervals for umbilical cord haematological parameters by Sex of Newborns from October to December, 2022, Mumbai, India (*n* = 127)

Parameter	Sex	N	Median	Mean	Max	Min	Upper	Lower	*P*-value
**WBC**	M	68	12.06	11.62	20.25	3.66	20.1	3.2	
	F	59	10.77	11.17	19.45	2.77	20.67	1.67	
	Mix	127	12.35	11.87	22.92	2.77	21.19	2.56	0.575
**RBC**	M	68	4.4	4.4	5.47	2.61	5.67	3.13	
	F	59	4.35	4.35	5.96	2.4	6.99	1.71	
	Mix	127	4.34	4.36	5.96	2.41	6.27	2.45	0.782
**Hb**	M	68	15.05	14.77	19.2	9	19.49	10.05	
	F	59	15.1	14.89	19.6	10.2	23.97	5.81	
	Mix	127	14.7	14.76	19.6	9	21.44	8.08	0.849
**HCT**	M	68	48.5%	48.2%	61.7%	34.8%	67.32%	29.1%	
	F	59	48.4%	47.71%	65.7%	34.96%	68.33%	27.08%	
	Mix	127	48%	48%	66%	33%	67%	29%	0.781
**MCV**	M	68	109.45	108.29	119.2	90.8	154.32	62.26	
	F	59	109.55	109.38	122.3	98.1	161.1	57.57	
	Mix	127	109.6	109.07	124.2	90.8	159.1	59.04	0.802
**MCH**	M	68	34.7	34.12	37.1	29.1	38.5	29.8	
	F	59	34.15	34.06	37.2	30.2	36.86	31.24	
	Mix	127	34.5	34.16	38	29.1	37.79	30.54	0.834
**MCHC**	M	68	31.5	31.5	32.8	29.2	32.82	30.1	
	F	59	31.3	31.15	32.2	28.6	32.73	29.58	
	Mix	127	31.3	31.31	32.8	28.6	32.75	29.87	**0.016**
**PLT**	M	68	257	254.4	350	112	493.6	15.27	
	F	59	244	247.25	356	42	493	0.69	
	Mix	127	249	248.21	356	42	479.46	16.97	0.739
**Neu%**	M	68	47.5%	48.9%	71.1%	5%	74.2%	23.5%	
	F	59	52%	51.6%	75.3%	26%	71.3%	32%	
	Mix	127	50%	50%	76%	6%	74%	26%	0.213
**Lym%**	M	68	40.8%	39.6%	63.9%	17%	62.17%	17.09%	
	F	59	38%	38.3%	64.6%	16%	57.5%	19.2%	
	Mix	127	38%	39%	67%	16%	62%	17%	0.482
**Eos%**	M	68	2.45%	2.49%	4.6%	0.6%	4.6%	0.33%	
	F	59	2.2%	2.2%	4.2%	0.5%	4%	0%	
	Mix	127	2.3%	2.4%	4.7%	0.3%	4.8%	0.1%	0.263
**Mon%**	M	68	7.05%	7.29%	11.5%	3.8%	11.1%	3.4%	
	F	59	7.5%	7.2%	11.5%	0.8%	11.5%	2.8%	
	Mix	127	7.3%	7.3%	11.5%	0.8%	11.4%	3.1%	0.775
**Bas%**	M	68	0.4%	0.42%	1%	0.2%	0.72%	0.14%	
	F	59	0.4%	0.41%	0.9%	0.2%	0.7%	0.1%	
	Mix	127	0%	0%	1%	0%	1%	0%	0.692
**Lym#**	M	68	4.45	4.44	7.19	1.74	7.84	1.03	
	F	59	4.21	4.22	11	1.1	8.05	0.4	
	Mix	127	4.21	4.27	7.21	0.2	5.97	2.58	0.302
**Eos#**	M	68	0.3	0.32	0.61	0	0.63	0.01	
	F	59	0.31	0.33	0.69	0.1	0.64	0.01	
	Mix	127	0.31	0.32	0.56	0.09	0.59	0.05	0.662
**Mon#**	M	68	0.84	0.87	1.98	0.11	1.67	0.072	
	F	59	0.76	0.78	1.45	0.03	1.47	0.05	
	Mix	127	0.84	0.83	1.62	0.03	1.57	0.09	0.3
**Bas#**	M	68	0.04	0.05	0.09	0.02	0.08	0.01	
	F	59	0.04	0.042	0.08	0.01	0.08	0	
	Mix	127	0.04	0.05	0.09	0.01	0.08	0	0.414
**Neu#**	M	68	5.91	5.98	11.3	1.22	10.77	1.18	
	F	59	5.38	5.8	12	1.43	11.23	0.36	
	Mix	127	5.86	6.08	14.24	1.22	8.7	3.4	0.693

Except %BAS, #BAS and #EOS, there was no statistical relevance observed in case of the age of mother.

The combined median and 95% reference value of cord blood parameters are shown in Table 3.

**Fig. 1:**
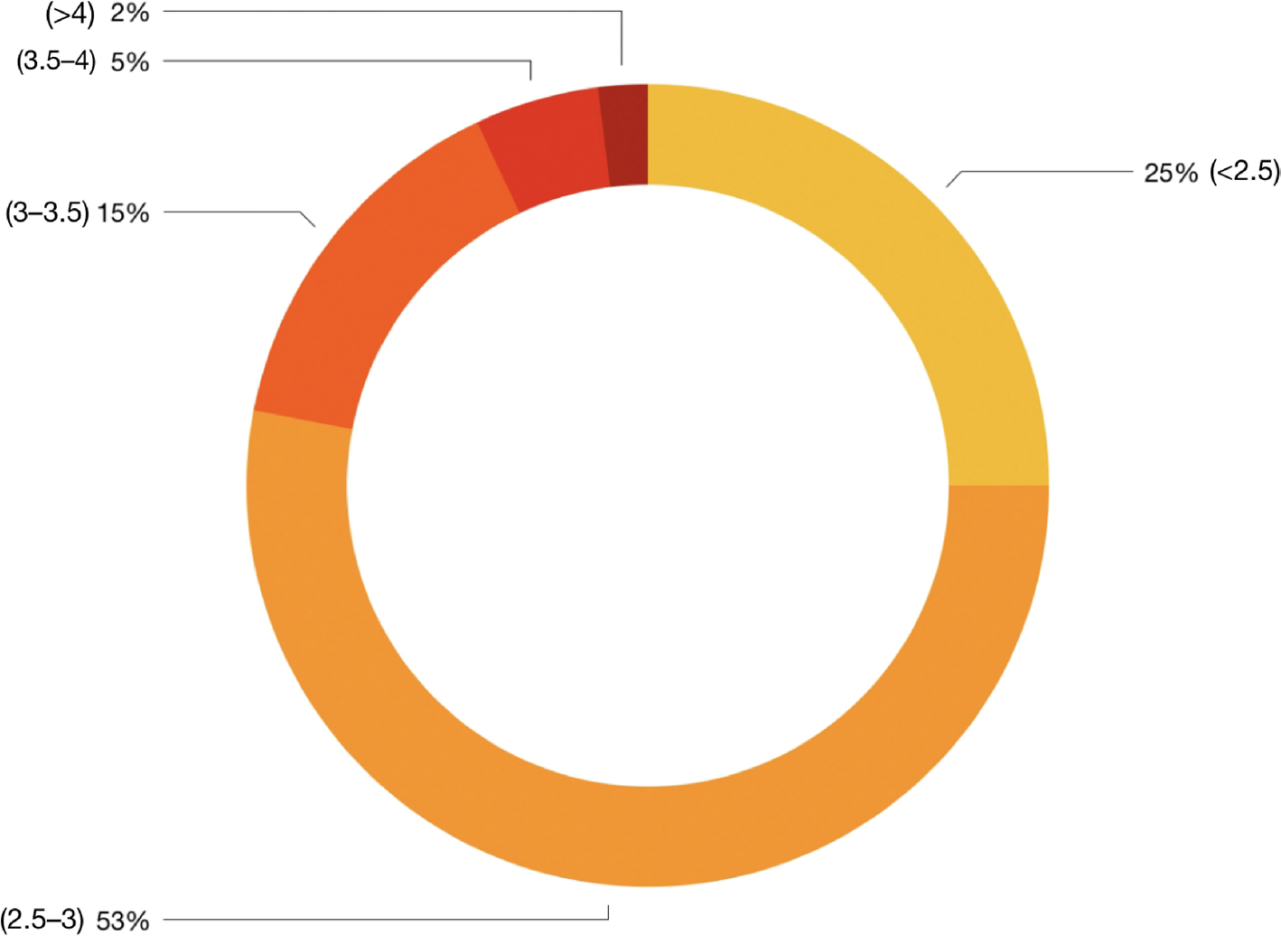
Percent distribution of total participants according to weights of infants in kg

The percentage distribution of total participants according to weights of infants in kg has been shown in Fig. 1.

All parameters except PLT and LYM# are elevated in the cord blood as compared to the venous blood of the neonate.

The study also provided a comparison between the established cord blood RI and that provided by Sysmex for 0–24 h old newborns as well as some other previous studies. Most studies have presented their findings as mean ± SD and hence the comparison made in this study was in accordance with them and has been displayed in Table 4.

**Table 4:** Mean ± SD or Median Comparison with company derived values, other published reference values and peripheral venous blood (PVB)

**Parameter**	**PVB **[1]	**Sysmex KX 21 **[21]	**Current Study**			**Sudan **[21]	**Nigeria **[20]
	**RI**	**RI**	**95% RI**	**Median**	**Mean ± SD**	**Mean ± SD**	**Mean ± SD**
**WBC**	5.75–13.5	9.0–30.0	2.56–21.19	12.35	11.87 ± 4.66	12.3 ± 4.17	13.1 ± 5.20
**RBC**	3–5.18	4.1–6.7	2.46–6.27	4.34	4.36 ± 0.95	4.34 ± 0.60	4.05 ± 0.55
**HBG**	9.3–12.9	15.0–24.0	8.08–21.44	14.7	14.76 ± 3.33	14.4 ± 1.55	13.9 ± 1.50
**HCT**	28–39	44–70	29–67	48	48 ± 9.0	44.1 ± 5.14	44.8 ± 5.78
**MCV**	72–98.6	102–115	59.04–159.1	109.6	109.07 ± 25	105.5 ± 5.14	110.4 ± 11.88
**MCH**	23.5–34	33.0–39.0	30.54–37.79	34.5	34.16 ± 1.81	33.5 ± 1.99	32.6 ± 4.13
**MCHC**	31.9–35.2	32.0–36.0	29.87–32.75	31.3	31.31 ± 0.72	33.1 ± 1.19	29.8 ± 1.64
**PLT**	253–552	140–385	16.97–479.46	249	248.21 ± 115.6	261 ± 83.16	225.1 ± 72.21
**LYM%**	-	-	17–62	38	39 ± 11	-	-
**NEU%**	-	-	26–74	50	50 ± 12	-	-
**NEU#**	0.7–5.8	6.0–26.0	3.4–8.7	5.86	6.08 ± 2.67	-	-
**LYM#**	1.96–8.94	2.3–10.8	2.58–5.97	4.45	4.27 ± 2.55	-	-

Units: WBC (× 10^9^/L), RBC (× 10^12^/L), HGB (g/dL), PLT (× 10^9^/L), MCV (fL), MCH (pg), MCHC (g/L)

**Fig. 2:**
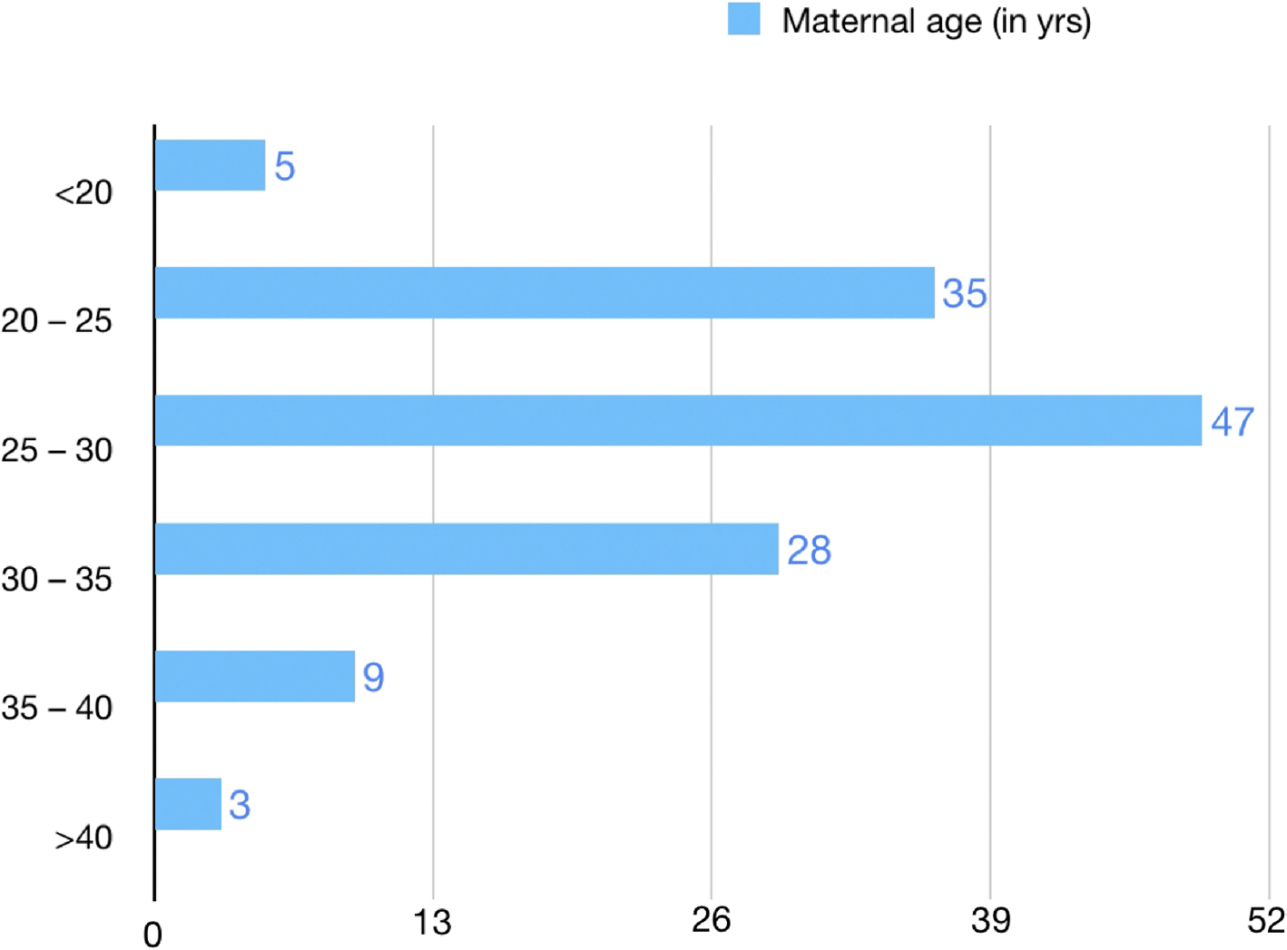
Distribution of data according to maternal age (*n* = 127)

A graphical distribution of the data according to maternal age has been shown in Fig. 2.

## Discussion

Providing reference intervals for selected haematological parameters from cord blood samples and establishing a comparison between these findings and company derived as well as published reports was the main aim of this study. A comparison of these results was done on the basis of sex of the infant, mode of delivery, age and obstetric history of the mother.

There was no statistically significant gender difference (p > 0.05) for all haematological parameters except that for MCHC. Previous studies from Greece [14], Korea, South India, Nepal, Saudi Arabia, Iran, Nigeria and Sudan [15–21] concluded that there are no statistically significant differences observed on the basis of gender of the infant. Although it was observed that the literature cannot provide a consistent observation regarding differences on the basis of gender in cord blood haematological parameters, the mechanisms yet remain to be well explored.

The current study demonstrated lowered RIs mainly for WBC, RBC, HGB, HCT, MCH, MCHC and absolute 2 part differential counts (#NEU, #LYM) parameters compared to reference value provided by Sysmex KX- 21 N haematology analyser for newborns (0–24 h). A remarkable difference was noted in the RI of absolute count of Neutrophils (3.4–8.7 versus 6.0–26.0 × 10^9^/L, current versus Sysmex RIs, respectively) [22]. A consistency with a study from South India [16] which reported a lower neutrophil count RI compared to the RI given by the company using the same machine Sysmex KX- 21 N corroborated the notion that neutropenia is a common condition in Indian subcontinent which may be associated with nutritional deficiencies of patients coming to government hospitals.

In the current study, MCV and PLT values were higher compared to Greece [14], Pakistan [23], Sudan [21], Nepal [17] and Adis Ababa [24]. High PLT could be in association with the fact that iron deficiency anaemia is widely prevalent in general population of India. MCV elevation may correlate to folate and cobalamin deficiency commonly seen in pregnant women. Although technical differences in the handling of the specimen, cord clumping time [25] and its further analysis [26] cannot be ruled out. It must also be noted that the current study has strictly followed all the protocols to ensure quality.

As discussed previously, the current study found lowered values of HCT and MCHC and elevated values of PLT and LYM# compared to Sysmex KX—21. It was also observed that these values showed a wide variation when compared to the studies carried out in southern part of India. Such inconsistencies reflect light upon the necessity for locally established RIs for the target population.

Physiological, socio-economic, geographical, nutritional, ethnic as well as maternal stress contribute to variations among populations. The differences seen in some studies could be associated with the stressful process of birth. The foetus suffers an inflammatory stress that elicits a change in the hemogram [27]. These haematological intervals for Indian cord blood may assist in efficient neonatal care and their management. It may also aid in improvising the Umbilical Cord Blood Banks all over India as well as Stem Cell Transplant which are not very commonly practised. It has thus been recommended that laboratories, stem cell transplant units, surgical units and all facilities associated with neonatal care to incorporate cord blood analysis into their routine testing as well as manuals and handbooks in line of improving neonatal care.

### Limitations of the study

The cases that were involved in the study were newborns who were born in only one hospital i.e. localized to one area. The reliability of the study would be increased if multiple locations could be studied. Ideally reference intervals should be separate depending on the ethnicity, gestational age, mode of delivery, etc. but this study generalizes the reference intervals without considering these factors. Another possible limitation can be the smaller sample size.

A comparison between the reference intervals of PVB and UCB has been provided in Table 4.

## Conclusions

Reference intervals play a vital role in guiding the assessment of haematological changes in neonatal care, but still there is no published reference interval for cord blood neonatal parameters from India. Although this study was conducted in a smaller population, we can still consider it due to the versatility in the background of people. However, the results need to be confirmed by larger samples from different parts of the country for wider use.

## Data Availability

All data generated or analysed during this study are included in this published article. The dataset can be available from the corresponding author on reasonable request.

## Abbreviations

RI: Reference interval
UCBC: Umbilical Cord Blood Collection
EDTA: Ethylenediaminetetraacetic acid
CBC: CompleteBlood Count
WBC: White Blood Cell
RBC: Red Blood Cell
HGB: Hemoglobin
HCT: Haematocrit
MCV: Mean Cell Volume
MCH: Meancell Hemoglobin
MCHC: Mean cell Hemoglobin Concentration
PLT: Platelet
LYM: Lymphocyte
MON: Monocytes
EOS: Eosinophils
BAS: Basophils
NEU: Neutrophils
CS: Cesarean section
SVD: Spontaneous Vaginal Delivery
